# Suppressing astrocytic GABA transaminase enhances tonic inhibition and weakens hippocampal spatial memory

**DOI:** 10.1038/s12276-025-01398-0

**Published:** 2025-02-03

**Authors:** Mingu Gordon Park, Jiwoon Lim, Daeun Kim, Won-Seok Lee, Bo-Eun Yoon, C. Justin Lee

**Affiliations:** 1 Center for Cognition and Sociality, Life Science Institute, Institute for Basic Science, Daejeon, South Korea; 2 IBS School, University of Science and Technology, Daejeon, South Korea; 3 Department of Molecular Biology, Dankook University, Cheonan, South Korea; 4 Institute of Tissue Regeneration Engineering, Dankook University, Cheonan, South Korea

**Keywords:** Astrocyte, Neurophysiology

## Abstract

Pharmacological suppression of γ-aminobutyric acid (GABA) transaminase (GABA-T), the sole GABA-degrading enzyme and a potential therapeutic target for treating brain disorders such as epilepsy, increases not only phasic inhibition but also tonic inhibition. However, the specific cellular source, neuromodulatory effects and potential therapeutic benefits of this enhanced tonic inhibition remain unexplored due to the lack of cell-type-specific gene manipulation studies. Here we report that the increase in tonic GABA currents observed after GABA-T suppression is predominantly due to increased tonic GABA release from astrocytes rather than action-potential-dependent synaptic GABA spillover. General GABA-T knockdown (KD) by a short hairpin RNA considerably increased tonic GABA currents in dentate granule cells, thereby enhancing tonic inhibition. An astrocyte-specific rescue of GABA-T following general GABA-T KD normalized the elevated tonic GABA currents to near control levels. Tetrodotoxin-insensitive tonic GABA currents were significantly increased after general GABA-T KD, whereas tetrodotoxin-sensitive tonic GABA currents showed no significant increase, suggesting that this enhanced tonic inhibition is primarily action-potential independent. General GABA-T KD reduced the spike probability of granule cells and impaired dorsal hippocampus-dependent spatial memory, which were fully reversed by astrocyte-specific GABA-T rescue. These findings suggest that suppressing astrocytic GABA-T may be sufficient to influence the excitatory/inhibitory balance in the brain and associated behaviors. Our study implies that the therapeutic benefits of pharmacological GABA-T suppression may be largely attributed to the modulation of astrocytic GABA-T and its impact on tonic GABA release from astrocytes.

**Figure:**
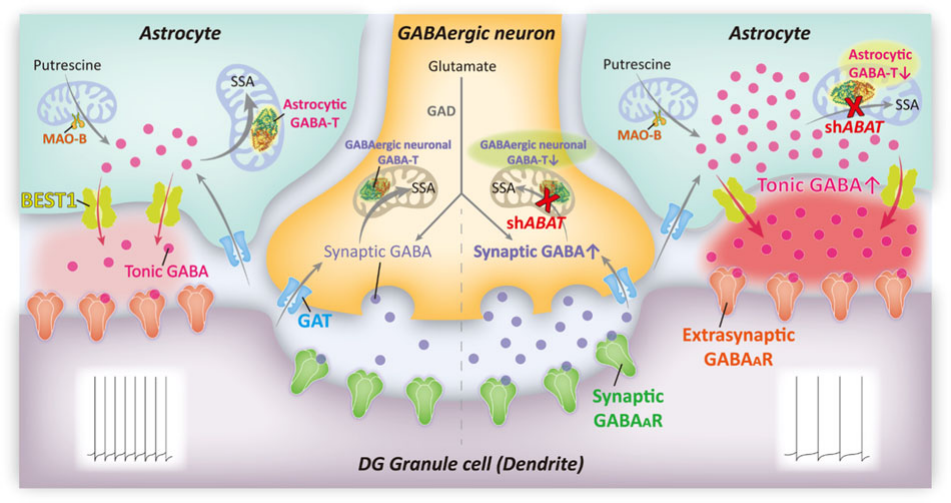
Here, we report distinct effects of GABA-T suppression depending on cell type; suppressing GABA-T in astrocytes enhances tonic inhibition, while its suppression in GABAergic neurons augments phasic inhibition. Our findings demonstrate that targeted suppression of astrocytic GABA-T not only enhances tonic GABA release from astrocytes but also significantly influences the excitation/inhibition balance in the brain, with consequential effects on behavior. This suggests that astrocytic GABA-T modulation holds promising potential for developing novel therapeutic strategies aimed at treating cognitive and neurological disorders through the regulation of astrocytic GABA metabolism. GAD glutamate decarboxylase, MAO-B monoamine oxidase B, BEST1 bestrophin 1, GABA-T GABA transaminase, GAT GABA transporter, DG dentate gyrus, SSA succinic semialdehyde.

Here, we report distinct effects of GABA-T suppression depending on cell type; suppressing GABA-T in astrocytes enhances tonic inhibition, while its suppression in GABAergic neurons augments phasic inhibition. Our findings demonstrate that targeted suppression of astrocytic GABA-T not only enhances tonic GABA release from astrocytes but also significantly influences the excitation/inhibition balance in the brain, with consequential effects on behavior. This suggests that astrocytic GABA-T modulation holds promising potential for developing novel therapeutic strategies aimed at treating cognitive and neurological disorders through the regulation of astrocytic GABA metabolism. GAD glutamate decarboxylase, MAO-B monoamine oxidase B, BEST1 bestrophin 1, GABA-T GABA transaminase, GAT GABA transporter, DG dentate gyrus, SSA succinic semialdehyde.

## Suppressing astrocytic GABA degradation enhances tonic inhibition in brain

This study explores how boosting a specific brain process called tonic inhibition affects memory and brain function. Tonic inhibition involves a chemical called γ-aminobutyric acid (GABA) that helps downregulate brain activity. Researchers wanted to understand how blocking GABA degradation in brain cells called astrocytes affects this process. The study focused on the hippocampus, a brain area important for memory. Researchers used mice to test their ideas. They reduced the expression of an enzyme called GABA transaminase in astrocytes, which normally helps break down GABA. This reduction increased astrocytic GABA release, enhancing tonic inhibition and reducing brain cell activity. They found that this change impaired long-term memory but not short-term memory. The results suggest that astrocytic GABA transaminase plays a crucial role in controlling brain activity and memory. By manipulating this enzyme, scientists might develop new treatments for neurological disorders.

This summary was initially drafted using artificial intelligence, then revised and fact-checked by the author.

## Introduction

Tonic inhibition, characterized by persistent inhibitory transmission that modulates neuronal excitability, results from the interaction between tonically released γ-aminobutyric acid (GABA) and high-affinity GABA_A_ receptors (GABA_A_Rs) located extrasynaptically^1–8^. Its importance is underscored by the observation that, in some cases, the charge conveyed by long-lasting tonic GABA currents can exceed that of short-lasting phasic GABA currents produced by presynaptically released GABA binding to synaptic GABA_A_Rs with low affinity^9^. Therefore, understanding the role of tonic inhibition in the brain can provide insights into various pathophysiological states, including neurodegenerative diseases, metabolic disorders, movement disorders, physical trauma and psychiatric disorders^10–24^, where the balance of neuronal excitation and inhibition may be compromised.

So far, the level of tonic inhibition has been readily reduced by suppressing either monoamine oxidase B (MAO-B)^10,13–16,18,20,24–32^, the key enzyme in the astrocytic GABA production pathway, or bestrophin 1^10,16,17,32–37^, the key ion channel mediating tonic GABA release from astrocytes. Through the suppression of MAO-B or bestrophin 1, impaired neuronal excitability can be restored in disease models, such as Alzheimer’s disease models^10,14^. By contrast, no studies have explored potential manipulations that increase tonic GABA release from astrocytes and tonic inhibition to suppress neuronal excitability, either under physiological conditions or for the treatment of brain disorders, such as epilepsy.

Elevating brain GABA levels is a key therapeutic strategy for counteracting excessive excitatory neurotransmission in many neurological and psychiatric disorders. To this end, suppression of the GABA degradation pathway has been proposed as a feasible and efficient method, particularly by inhibiting GABA transaminase (GABA-T), which is the only enzyme responsible for GABA degradation in mammals^38^. This enzyme, encoded by the *ABAT* gene, breaks down GABA that is either produced or taken up by astrocytes and GABAergic neurons^17,25,32,39^. GABA-T transforms GABA into succinic semialdehyde (SSA) while simultaneously converting α-ketoglutarate into glutamate. A downstream enzyme called SSA dehydrogenase (SSADH), encoded by the *ALDH5A1* gene, subsequently converts SSA into succinate. Previous studies have primarily focused on GABAergic neurons, where pharmacological GABA-T suppression increases GABA accumulation and synaptic GABA release, changes that are expected to dampen excessive neuronal activity and provide therapeutic benefits for epilepsy and drug addiction^40–45^. However, although several studies have highlighted a substantial increase in tonic inhibition due to a disruption of the GABA degradation pathway^7,46–49^, in-depth research into its neuromodulatory and therapeutic impacts is still lacking, largely because the cellular origin of the enhanced tonic inhibition remains ambiguous.

In the present study, we investigate whether astrocytic GABA-T is responsible for enhanced tonic inhibition upon pharmacological GABA-T suppression. We focused on the dorsal hippocampal dentate gyrus (DG), a region that is not only crucial for spatial learning and memory but also susceptible to modulation by tonic inhibition^10,14,22^. We found that astrocyte-specific suppression of GABA-T can effectively increase astrocytic tonic GABA release and tonic inhibition, thereby controlling neuronal excitability and related behaviors.

## Materials and methods

### Animals

Male C57BL/6J mice (8–16 weeks old) obtained from the Institute for Basic Science (IBS) Research Solution Center were used. All the animals were maintained on a 12 h light‒dark cycle in a specific-pathogen-free facility with controlled temperature and humidity and had free access to food and water. All experimental procedures were conducted according to protocols approved by the Institutional Animal Care and Use Committee of the IBS. Every effort was made to minimize the animals’ suffering and reduce the number of animals used.

### Viruses

The following viruses were used for this study: Lenti-pSicoR-shSCR-GFP, Lenti-pSicoR-sh*ABAT*-GFP, AAV_5_-pSicoR-shSCR-mCherry, AAV_5_-pSicoR-sh*ABAT*-mCherry, AAV_5_-gfaABC1D-GFP-myc-Cre, AAV_5_-gfaABC1D-GFP and AAV_5_-gfaABC1D-m*ABAT*-2A-GFP. For the control short hairpin RNA (shRNA) (scrambled shRNA, shSCR), a scrambled sequence (5′-TCGCATAGCGTATGCCGTT-3′) was inserted in place of the sh*ABAT* sequence.

### Primary cultures

Primary astrocytes were obtained from C57BL/6J mouse pups at postnatal days 0 to 2 (P0–2). Due to the very young age of the pups, it was not feasible to determine the sex of the pups from which the cells used in the culture were generated. The hippocampus was carefully dissected and finely chopped. The tissue was then gently triturated with a Pasteur pipette to achieve a single-cell suspension. The dissociated cells were then seeded on 35 mm culture dishes precoated with poly-d-lysine (0.1 mg/ml). The cells were grown in DMEM supplemented with 25 mM glucose, 2 mM glutamine, 10% heat-inactivated horse serum, 10% heat-inactivated fetal bovine serum and 1% penicillinn‒streptomycin. On the third day of culture, the cells were washed with PBS through repeated pipetting, and the medium was then replaced to remove debris and floating cells. The cultured cells were maintained at 37 °C in a humidified incubator with 5% CO_2_.

### Immunocytochemistry

The cultured primary hippocampal astrocytes were seeded onto 12 mm circular cover glasses (Paul Marienfeld, 111520) and treated with 150 μM vigabatrin (VGB, Tocris, 0808) for 24 h. After treatment, the samples were fixed with 4% paraformaldehyde (PFA) in 0.1 M PBS for 15 min. The samples were then washed three times with PBS and incubated for 1 h in a blocking solution containing 0.3% Triton-X and 2% donkey serum in PBS. The samples were subsequently immunostained with a chicken polyclonal antibody against glial fibrillary acidic protein (GFAP) (1:500, Millipore, AB5541) and guinea pig polyclonal antibody against GABA (1:200, Millipore, AB175), both of which were mixed into the blocking solution. The samples were incubated with the primary antibodies at 4 °C for 24 h with gentle rocking. Afterward, the samples were washed three times with PBS and then incubated with an Alexa Fluor 488-conjugated donkey anti-chicken polyclonal antibody (1:500, Jackson ImmunoResearch, 703-545-155) and an Alexa Fluor 594-conjugated donkey anti-guinea pig polyclonal antibody (1:500, Jackson ImmunoResearch, 706-585-148), both of which were mixed with the blocking solution. The secondary antibody incubation was performed at room temperature for 2 h with gentle rocking. Afterward, the samples were washed three times with PBS. During the second round, DAPI (1:1,000, Thermo Fisher, 62248) was added to the PBS to visualize the nuclei of the cells. Finally, the cover glasses were mounted with fluorescence mounting medium (Agilent, S3023) and left to dry. The fluorescence images were captured using a Zeiss LSM900 confocal microscope, and *Z*-stack images were processed with ZEN Blue (Zeiss, version 3.2) and ImageJ (version 1.54f).

### RT‒PCR

The total RNA was extracted from the collected cell pellet using an RNeasy Mini Kit (Qiagen, 74104). The extracted RNA was then converted to complementary DNA using the SuperScript III First-Strand Synthesis System (Invitrogen, 18080051). Subsequently, we performed PCR using QuestTaq PCR premix (Zymo Research, E2051). The procedure started with a 1 min denaturation phase at 95 °C, followed by 30 cycles of the following sequence: denaturation at 95 °C for 30 s, annealing at 55 °C for 30 s and extension at 72 °C for 10 s. Then, a final extension was performed at 72 °C for 7 min. All steps were optimized on the basis of the manufacturers’ protocols. The sequences of the primers used for RT‒PCR were as follows:

mouse *ABAT* forward, 5′-TGGTACCGGAGTAAGGAACG-3′;

mouse *ABAT* reverse, 5′-GAGTGTGTGGTCGCTAAGCA-3′;

mouse *GAPDH* forward, 5′-ACCCAGAAGACTGTGGATGG-3′;

mouse *GAPDH* reverse, 5′-CACATTGGGGGTAGGAACAC-3′.

### Western blot

The protein samples were separated on 10% SDS‒PAGE gels under reducing conditions and then electrophoretically transferred onto polyvinylidene difluoride (PVDF) membranes using the iBlot2 Dry Blotting System (Thermo Fisher Scientific). After an incubation with 5% skim milk in TBST (10 mM Tris, pH 8.0; 150 mM NaCl; 0.5% Tween 20) for 1 h at 25 °C, the membranes were washed once with TBST and incubated with a rabbit monoclonal antibody against GABA-T (1:1,000, Abcam, ab108249) or a rabbit monoclonal antibody against β-actin (1:1,000, Abcam, ab133626) at 4 °C overnight. The membranes were subsequently washed with TBST five times and incubated with horseradish peroxidase-conjugated goat anti-rabbit IgG (1:3,000, BioActs, RSA1221) for 1 h at 25 °C. The membranes were subsequently washed with TBST five times and developed with an enhanced chemiluminescence (ECL) substrate (Bio-Rad, 1705061). The images of chemiluminescence were captured with an ImageQuant LAS 500 chemiluminescence CCD camera (Cytiva).

### Stereotaxic injection

The mice were anesthetized with vaporized isoflurane (induction 3–4%, maintenance 1.5–2%) and placed into stereotaxic frames (RWD). The scalp was incised and a hole was drilled into the skull above the hippocampal DG (anterior/posterior, −1.8 mm from the bregma; medial/lateral, ±1.2 mm from the bregma; dorsal/ventral, −1.9 mm from the skull). The virus was loaded into a 33G blunt NanoFil needle (WPI, NF33BL) and injected bilaterally into the DG at a rate of 0.2 μl/min for 5 min (total 1 μl) using a syringe pump (KD Scientific). At the end of the virus infusion, the position of the needle was maintained in the brain for another 10 min to minimize backflow of the virus. All the viruses used were produced by the virus facility of the IBS. A total of 6 weeks after the virus injection, the mice were subjected to ex vivo patch-clamp recordings and in vivo experiments.

### Immunohistochemistry

The mice were anesthetized via an intraperitoneal injection of alfaxan (80 mg/kg) and xylazine (20 mg/kg) dissolved in saline. Then, the mice were perfused with saline followed by an ice-cold 4% PFA solution. The extracted brains were postfixed overnight at 4 °C in a PFA solution, transferred to a 30% sucrose solution and incubated at 4 °C for 48 h. The coronal hippocampal sections with a thickness of 30 μm were prepared using a cryotome and stored in storage solution at 4 °C. The sections were washed three times with 0.1 M PBS and incubated for 1 h with a blocking solution (0.3% Triton-X-100, 2% donkey serum in 0.1 M PBS). The sections were subsequently incubated for 24 h at 4 °C with the required concentrations of primary antibodies mixed with the blocking solution under gentle agitation. The primary antibodies used for GABA-T immunostaining in astrocytes were a chicken polyclonal antibody against GFAP (1:500, Millipore, AB5541) and a rabbit monoclonal antibody against GABA-T (1:500, Abcam, ab216465). The sections were washed three times with PBS to remove unbound primary antibodies. After washing, the sections were incubated with secondary antibodies for 2 h at room temperature. The secondary antibodies used for GABA-T immunostaining in astrocytes were donkey anti-chicken Alexa 405 or 594 (1:500, Jackson ImmunoResearch, 703-475-155 or 703-585-155) and donkey anti-rabbit Alexa 647 (1:500, Jackson ImmunoResearch, 711-605-152). Afterward, the sections were washed three more times with PBS to remove unbound secondary antibodies. The sections were mounted with fluorescence mounting medium (Agilent, S3023) and dried. A series of fluorescence images were obtained using a Zeiss LSM900 confocal microscope. Z-stack images were processed with ZEN Blue (Zeiss, version 3.2) and ImageJ (version 1.54 f).

### Slice preparation

The mice were anesthetized with vaporized isoflurane before being decapitated to isolate the brain. The isolated brains were then excised and submerged in an ice-cold sucrose slicing solution containing: sucrose 212.5 mM, KCl 3 mM, NaHCO_3_ 26 mM, NaH_2_PO_4_ 1.25 mM, CaCl_2_ 0.1 mM, MgCl_2_ 5 mM and glucose 10 mM. Next, each brain was glued onto the stage of a vibrating microtome (DSK, Linear Slicer Pro7), and 300-μm-thick coronal slices were prepared. For stabilization, the slices were incubated at room temperature for at least 30 min in an artificial cerebrospinal fluid (ACSF) solution with the following composition: NaCl mM 124, KCl 3 mM, NaHCO_3_ 24 mM, NaH_2_PO_4_ 1.25 mM, CaCl_2_ 2 mM, MgCl_2_ 1 mM and glucose 10 mM. All the solutions were continuously gassed with a mixture of 95% O_2_ and 5% CO_2_.

### Tonic GABA current recordings

The slices were transferred to a recording chamber that was continuously perfused with the ACSF solution (flow rate 2 ml/min). The slice chamber was mounted on the stage of an upright microscope (Nikon) and viewed with a 60× water immersion objective (numerical aperture (NA) 1.00, working distance (WD) 2.80) using infrared differential interference contrast optics. The cellular morphology was visualized with an sCMOS camera (Hamamatsu) and Imaging Workbench software (INDEC BioSystems). Whole-cell voltage-clamp recordings were performed on granule cell (GC) somata located in the outer molecular layer of the dorsal hippocampal DG. The holding potential was −70 mV. The patch pipettes (resistance of 6–8 MΩ) were filled with an internal solution containing the following: CsCl 135 mM, NaCl 4 mM, CaCl_2_ 0.5 mM, HEPES buffer 10 mM, EGTA 5 mM, Mg-ATP 2 mM, Na_2_-GTP 0.5 mM and QX-314 10 mM. The pH was adjusted to 7.2 with CsOH, and the osmolality was measured as 278–285 mOsmol/kg. The baseline current was stabilized with a bath application of D-AP5 (50 μM, Tocris, 0106) and CNQX (20 μM, Tocris, 0190) to isolate GABA_A_R currents from NMDA and AMPA currents. Electrical signals were digitized and sampled using a Digidata 1440A digitizer and MultiClamp 700B amplifier with pClamp10.2 software (Molecular Devices). The raw data were filtered at 2 kHz. The tonic GABA currents were measured by the baseline shift in response to the bath application of tetrodotoxin (TTX) (1 μM, Alomone Labs, T-550) or bicuculline (50 μM, Tocris, 0109) using Clampfit software (version 10.7). The frequency and amplitude of spontaneous inhibitory postsynaptic currents (sIPSCs) before the bicuculline treatment were detected and measured with MiniAnalysis software (Synaptosoft).

### Evoked spike probability

Synaptically evoked spikes in GCs were generated by a 0.1 Hz stimulation of lateral perforant path fibers (100 μs duration; intensities ranging from 100 to 1000 μA in 100 μA increments) using a tungsten bipolar electrode connected to a stimulus isolator (WPI). The evoked excitatory postsynaptic potentials were recorded using glass pipette electrodes (resistance of 6–8 MΩ) filled with an intracellular solution containing the following: K-gluconate 120 mM, KCl 20 mM, NaCl 2 mM, HEPES 20 mM, EGTA 0.5 mM, glucose 10 mM, Mg-ATP 2 mM and Na_2_-GTP 0.5 mM (pH 7.2 adjusted with KOH). The electrical signals were digitized and sampled using a Digidata 1440A digitizer and MultiClamp 700B amplifier with pClamp10.2 software (Molecular Devices). The raw data were filtered at 2 kHz. The spike probability was calculated as the ratio of the number of successful (spike-generating) stimuli to the total number of stimulations.

### Y-maze test

Each mouse was placed in the center of a symmetrical Y-maze with three identical arms, each 30 cm long and 16 cm high. It was given free access to the arms for a 10 min session. The test was video-recorded using EthoVision XT (Noldus), and the results, including the total number of arm entries and alternation behavior, were analyzed later. The percentage of spontaneous alternation was calculated as follows:

Spontaneous alternations (%) = number of spontaneous alternations/(total number of arm entries − 2) × 100.

An entry was counted only when all four limbs of the mouse were inside the arm.

### Novel place recognition test

Each mouse was habituated to an open field (40 × 40 cm with 40 cm high walls). Each mouse was exposed to two identical objects for a brief period (10 min) in the open field, with a simple visual cue placed on the wall to provide a spatial reference. The time spent exploring each object was recorded during the acquisition trial. After 1 h in the home cage, one of the familiar objects was moved to a novel place in the same arena. The mouse was then exposed to both the unchanged object and the relocated object for 10 min during the retrieval trial. Memory was measured as the proportion of time the animal spent exploring the relocated object versus the unchanged object. The discrimination index was calculated as follows:

Discrimination index (%) = (time exploring the newly placed object − time exploring the familiar object)/(time exploring the newly placed object + time exploring the familiar object) × 100.

As some mice exhibited a biased preference for contacting one of the two identical objects during the acquisition trial, changes in the discrimination index from the acquisition trial to the retrieval trial were calculated.

### Statistical analysis

For data presentation and statistical analyses, we used GraphPad Prism (GraphPad, version 10.0.2). When comparing two groups, either the unpaired or paired *t*-test was used if the data passed both the normality test and the equal variance test. If the data from two groups did not pass the normality test, the Mann‒Whitney test was used. If the data from two groups passed the normality test but failed the equal variance test, Welch’s *t*-test was employed. For comparisons involving more than two groups, we used ordinary one-way analysis of variance (ANOVA) with Tukey’s post hoc test when the data passed both the normality test and the equal variance test. When the data did not pass the normality test, the Kruskal‒Wallis test with Dunn’s post hoc test were used. For comparisons with two independent variables, we used two-way ANOVA with Šídák’s multiple comparisons test for the post hoc analysis to correct for multiple comparisons. For repeated-measures data, we applied a two-way repeated-measures ANOVA with uncorrected Fisher’sleast significant difference (LSD), allowing more sensitive post hoc comparisons without correction for multiple comparisons.

## Results

### Silencing the GABA-T gene increases both phasic and tonic inhibition in dentate GCs

**Fig. 1:**
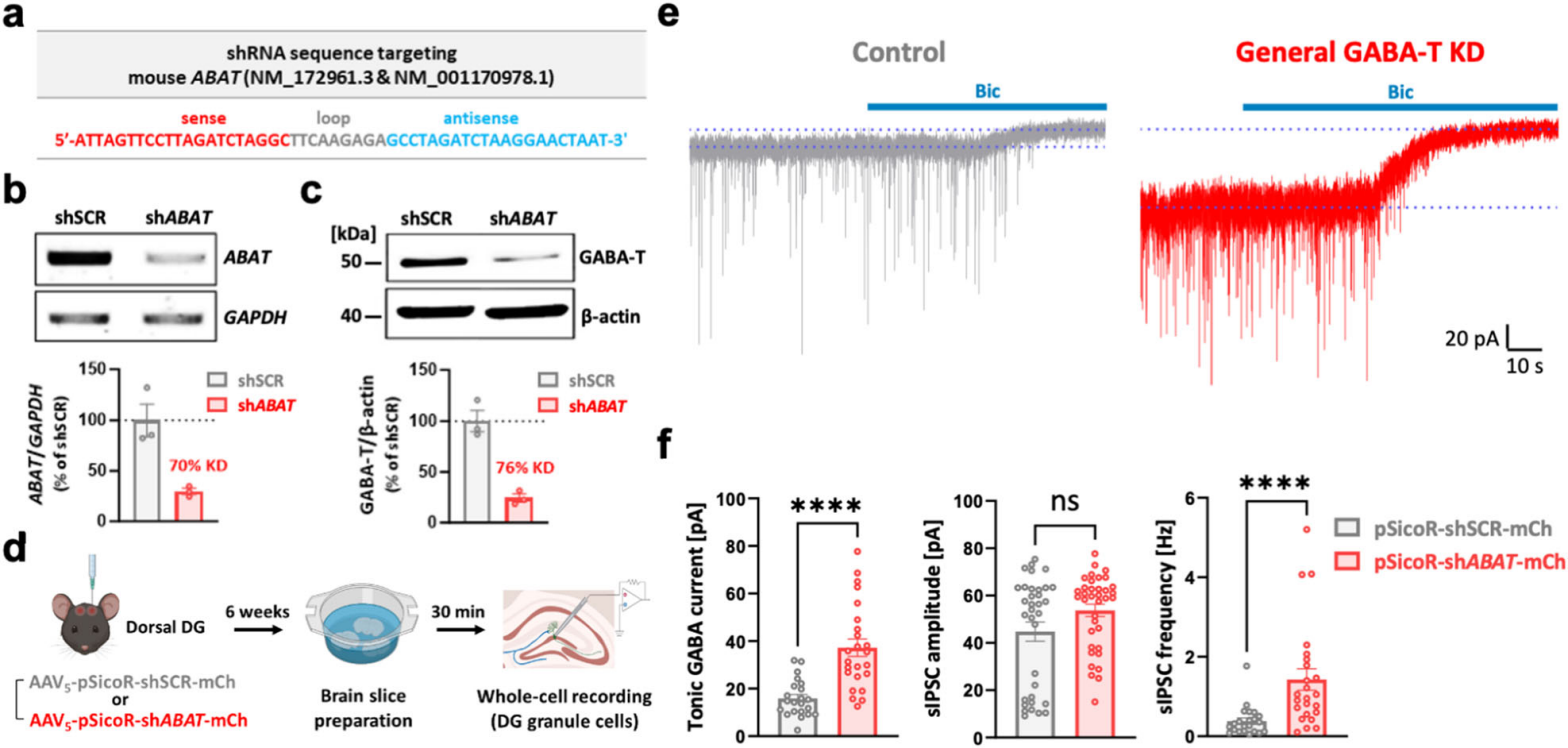
Non-cell-type-specific suppression of GABA-T enhances both phasic and tonic inhibition in dentate GCs. **a**, Mouse *ABAT* shRNA (sh*ABAT*) sequence (the sense in red, loop in gray and antisense in blue). **b**, Top: RT‒PCR analysis of *ABAT* and *GAPDH* mRNA levels in cultured mouse hippocampal astrocytes infected with either Lenti-pSicoR-shSCR-GFP or Lenti-pSicoR-sh*ABAT*-GFP. Bottom: comparative bar graphs presenting the ratio of *ABAT*/*GAPDH* expression. Each dot indicates a different culture batch. **c**, Top: western blot analysis of GABA-T- and β-actin-immunoreactive proteins from cultured mouse hippocampal astrocytes infected with either Lenti-pSicoR-shSCR-GFP or Lenti-pSicoR-sh*ABAT*-GFP. Bottom: comparative bar graphs presenting the ratio of GABA-T/β-actin expression. Each dot indicates a different culture batch. **d**, A workflow illustrating the nonspecific silencing of the GABA-T gene in the dorsal hippocampal DG, followed by brain slice preparation and whole-cell voltage-clamp recordings in GCs. **e**, Representative traces of GABA_A_R-mediated phasic and tonic GABA currents from the control (shSCR) and general GABA-T KD (sh*ABAT)* groups. **f**, A comparative bar graphs of the tonic GABA current (left), sIPSC amplitude (middle) and sIPSC frequency (right). sIPSCs recorded before the bicuculline (Bic) treatment were analyzed. *n* = 5 mice per group. The data are presented as the mean ± standard error of the mean. The individual dots refer to cells, unless otherwise specified. The *P* values were obtained via Welch’s *t*-test for **f** (left) as well as the Mann‒Whitney test (middle and right). ****P < 0.0001, nonsignificant (ns) indicates a *P* > 0.05.

We designed and developed a shRNA to genetically suppress the expression of *ABAT* in mice and to recapitulate the increases in both phasic and tonic GABA-mediated inhibition observed with pharmacological GABA-T suppression (Fig. 1a). We treated cultured mouse hippocampal astrocytes with Lenti-pSicoR-*ABAT* shRNA (sh*ABAT)*-GFP and subsequently evaluated the knockdown (KD) efficiency through RT‒PCR and western blot analyses. These analyses revealed KD efficiencies of 70% and 76%, respectively, with sh*ABAT* (Fig. 1b,c). Using this sh*ABAT*, we aimed to achieve a non-cell-type-specific KD of GABA-T in the dorsal hippocampal DG to measure phasic and tonic GABA currents. To this end, we administered AAV_5_-pSicoR-sh*ABAT*-mCherry via injection at specified coordinates mediolateral [ML] ±1.2, anteroposterior [AP] −1.8, dorsoventral [DV] −1.9. The KD efficiency of the sh*ABAT* virus in dorsal hippocampal tissue was 81%, which was comparable with the KD efficiency observed in cultured astrocytes (Fig. 1c and Supplementary Fig. 1a). A total of 6 weeks after the injection, we prepared hippocampal slices and performed whole-cell voltage-clamp recordings on GCs within this region (Fig. 1d). As expected, the general GABA-T KD group presented significantly greater tonic GABA currents than did the control group treated with the shSCR (Fig. 1e,f). While the amplitude of sIPSCs remained unaffected, we observed a considerable increase in the frequency of sIPSCs after exposure to sh*ABAT* (Fig. 1e,f). These findings underscore that the general GABA-T KD using the sh*ABAT* can readily enhance both phasic and tonic GABA inhibition.

### Astrocytic GABA-T mediates GABA clearance

**Fig. 2:**
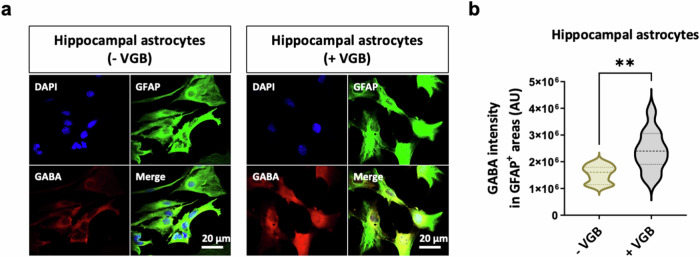
Astrocytic GABA-T is critical for GABA clearance. **a**, Immunostaining for GFAP and GABA in cultured mouse hippocampal astrocytes cultured in the absence or presence of 150 μM VGB for 24 h. **b**, Violin plots showing the average intensity of GABA staining in GFAP^+^ areas. The data are presented as the mean ± standard error of the mean. The *P* value was obtained via Welch’s *t*-test for **b**. **P < 0.01.

Before examining the link between astrocytic GABA-T and tonic inhibition, the pivotal function of astrocytic GABA-T in the regulation of GABA clearance must be confirmed. Therefore, we treated cultured mouse hippocampal astrocytes with VGB, the first Food and Drug Administration-approved GABA-T inhibitor^50^. Then, we performed immunocytochemistry for GFAP and GABA, as previously described^26,51^. As a result, we observed a clear increase in the GABA intensity within GFAP^+^ areas upon VGB (150 μM) treatment (Fig. 2a,b). These findings indicate that astrocytic GABA-T plays an important role in regulating intracellular GABA levels.

### Suppressing GABA-T in astrocytes leads to increased tonic inhibition but not phasic inhibition in dentate GCs

**Fig. 3:**
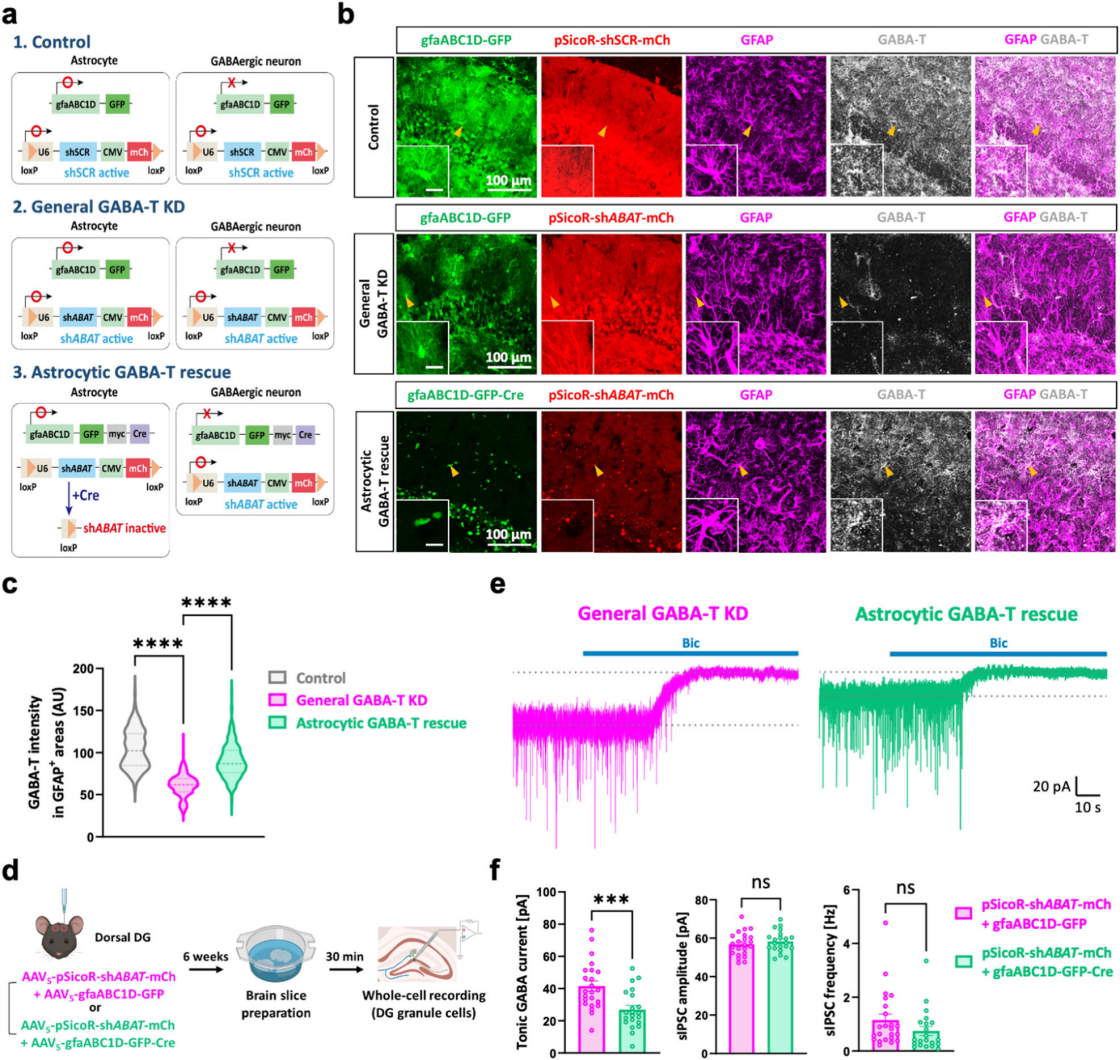
Astrocytic GABA-T selectively modulates tonic inhibition but not phasic inhibition in dentate GCs. **a**, A scheme of the Cre-dependent termination of sh*ABAT* expression specifically in astrocytes. **b**, Immunostaining for GABA-T and GFAP in the control, general GABA-T KD and astrocytic GABA-T rescue groups. The GFP–Cre signal is localized in the nucleus, and the mCherry (mCh) signal indicates the expression of the shRNA. The magnified inset image shows the astrocyte indicated by the yellow arrowhead (inset scale bar, 20 μm). **c**, The violin plots comparing the average intensity of GABA-T staining in the GFAP^+^ areas among the three groups. **d**, A workflow illustrating the silencing of the GABA-T gene (either including or excluding astrocytes) in the dorsal hippocampal DG, followed by the brain slice preparation and whole-cell voltage-clamp recordings in GCs. **e**, The representative traces of GABA_A_R-mediated phasic and tonic GABA currents from the general GABA-T KD and astrocytic GABA-T rescue groups. **f**, Comparative bar graphs of the tonic GABA current (left), sIPSC amplitude (middle) and sIPSC frequency (right). sIPSCs recorded before the bicuculline (Bic) treatment were analyzed. *n* = 5 mice per group. The data are presented as the mean ± standard error of the mean. The individual dots refer to cells, unless otherwise specified. The *P* values were obtained via the Kruskal‒Wallis test for **c** and an unpaired *t*-test for **f** (left and middle), as well as a Mann‒Whitney test for **f** (right). ***P < 0.001, ****P < 0.0001; nonsignificant (ns) indicates a *P* > 0.05.

Traditional irreversible GABA-T inhibitors, such as gabaculine and VGB, have been shown to increase both the intracellular and extracellular GABA levels in cultured human astrocytes^52^. This intriguing observation, along with our data (Fig. 2a,b), led to the hypothesis that astrocytic GABA-T could be an important contributor to the observed increase in tonic GABA currents after pharmacological GABA-T inhibition. We investigated this hypothesis by performing a general GABA-T KD, followed by an astrocyte-specific GABA-T rescue achieved through genetic manipulations. This approach was necessary because pharmacological GABA-T inhibition is not cell-type specific. By coinjecting AAV_5_-pSicoR-sh*ABAT*-mCherry and AAV_5_-gfaABC1D-GFP-myc-Cre into the dorsal hippocampal DG^53,54^, we were able to achieve Cre-dependent termination of sh*ABAT* expression exclusively in astrocytes (Fig. 3a,d). Unlike green fluorescent protein (GFP), which was localized in the cytosol of astrocytes, the Cre-conjugated GFP (GFP–Cre) fusion protein was localized in the cell nucleus of astrocytes because of its nuclear localization signal peptide (Fig. 3b). We performed immunohistochemistry with antibodies against GFAP and GABA-T to verify the changes in astrocytic GABA-T levels as a result of astrocyte-specific GABA-T manipulation (Fig. 3b). We found that the general GABA-T KD significantly reduced the levels of GABA-T in astrocytes. Moreover, this reduction in astrocytic GABA-T levels was reversed by rescue of astrocytic GABA-T (Fig. 3b,c), highlighting the efficacy and specificity of our genetic manipulation. Next, we conducted whole-cell voltage-clamp recordings on GCs to measure both tonic and phasic GABA-mediated currents at 6 weeks after the virus injection (Fig. 3d). Upon the astrocyte-specific rescue of GABA-T, the tonic GABA currents in GCs, which were elevated due to the general GABA-T KD, nearly returned to the control levels (Fig. 3e,f). This observation suggests that the suppression of astrocytic GABA-T selectively increases tonic inhibition. Notably, the amplitude and frequency of sIPSCs remained unchanged, indicating that astrocytic GABA-T is not involved in modulating phasic inhibition in GCs (Fig. 3f).

### The overexpression of GABA-T in astrocytes reduces tonic inhibition in dentate GCs

**Fig. 4:**
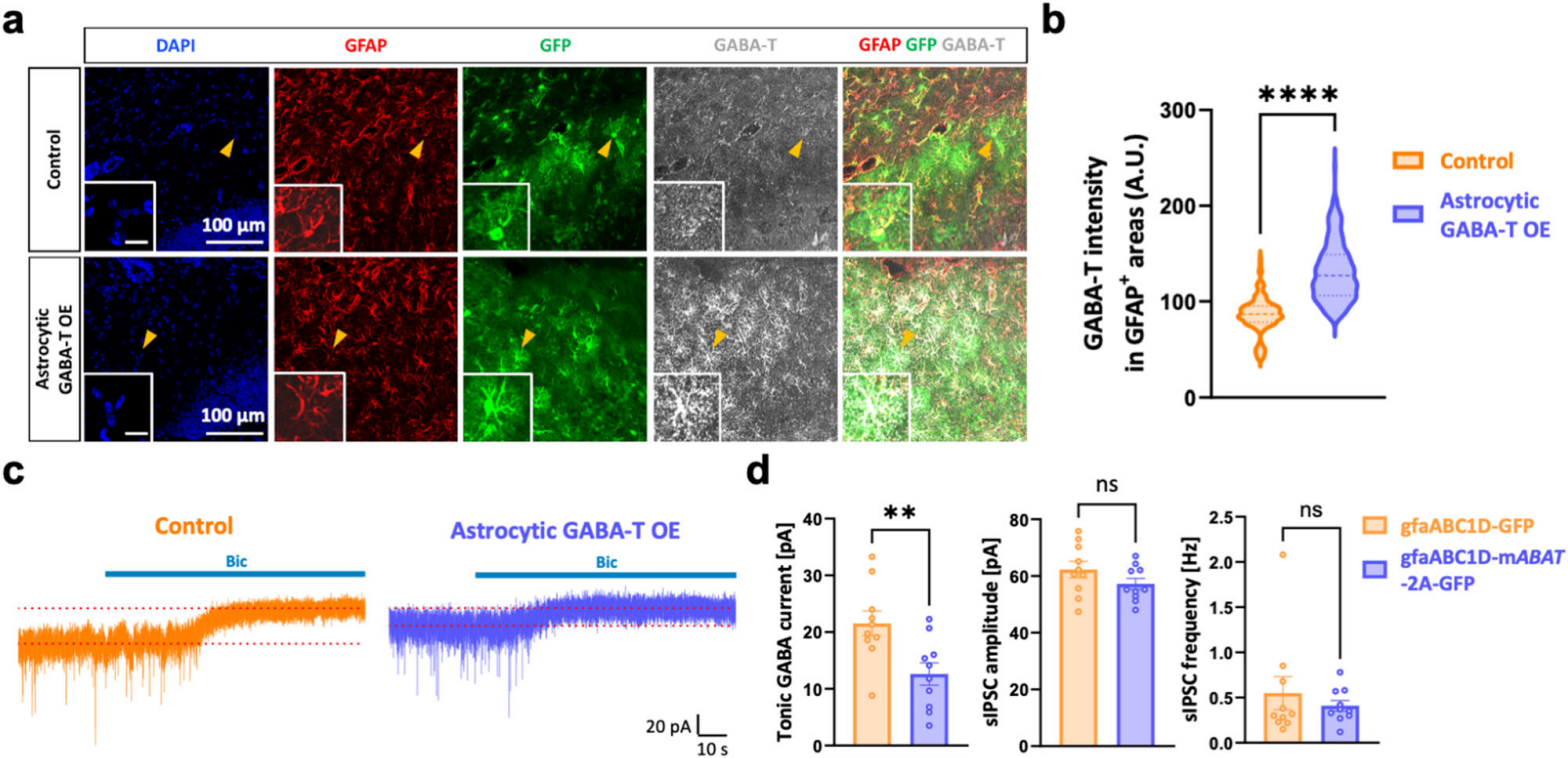
Astrocytic GABA-T overexpression is sufficient to increase GABA clearance and reduce tonic inhibition in dentate GCs. **a**, Immunostaining for GABA-T and GFAP in the control and astrocytic GABA-T-overexpressing (OE) groups. The GFP signal indicates the expression of the virus. The magnified inset image shows the astrocyte indicated by the yellow arrowhead (inset scale bar, 20 μm). **b**, Violin plots comparing the average intensity of GABA-T staining in GFAP^+^ areas between the two groups. **c**, Representative traces of GABA_A_R-mediated phasic and tonic GABA currents from the control and astrocytic GABA-T OE groups. **d**, Comparative bar graphs of the tonic GABA current (left), sIPSC amplitude (middle) and sIPSC frequency (right). sIPSCs recorded before the bicuculline (Bic) treatment were analyzed. *n* = 3 mice per group. The data are presented as the mean ± standard error of the mean. The individual dots refer to cells, unless otherwise specified. The *P* values were obtained via unpaired *t*-tests for **d** (left and middle) and Mann‒Whitney tests for **b** and **d** (right). **P < 0.01, ****P < 0.0001; nonsignificant (ns) indicates a *P* > 0.05.

We specifically overexpressed mouse *ABAT* (m*ABAT*) in astrocytes by injecting AAV_5_-gfaABC1D-m*ABAT*-2A-GFP into the dorsal hippocampal DG to further investigate the causal relationship between astrocytic GABA-T and tonic inhibition (Fig. 4a,b). This approach resulted in a 3.7-fold increase in GABA-T levels compared with those in the control (Supplementary Fig. 1b). Compared with basal levels of tonic inhibition, astrocytic m*ABAT* overexpression reduced tonic inhibition in GCs (Fig. 4c,d). However, the amplitude and frequency of sIPSCs were unaltered by astrocytic m*ABAT* overexpression (Fig. 4c,d). Collectively, these results reveal that astrocytic GABA-T strongly impacts the degradation of GABA, thereby playing an important role in regulating tonic inhibition.

### Synaptic GABA spillover does not contribute primarily to the enhanced tonic inhibition observed upon GABA-T suppression in dentate GCs

**Fig. 5:**
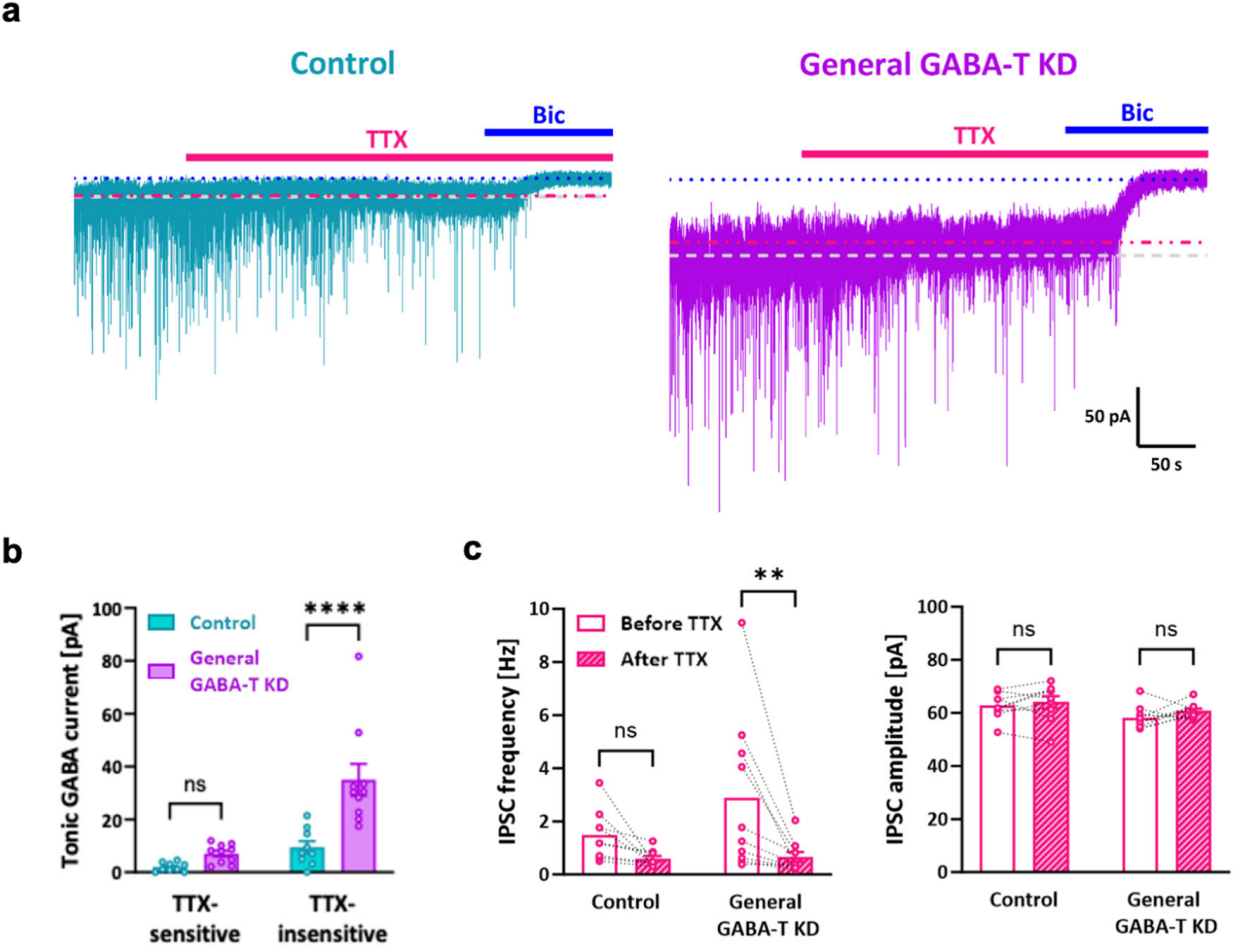
Enhanced tonic inhibition due to GABA-T suppression is not driven primarily by synaptic GABA spillover in dentate GCs. **a**, Representative traces of GABA_A_R-mediated phasic and tonic GABA currents from the control and general GABA-T KD groups treated with TTX and bicuculline (Bic). TTX-sensitive tonic GABA currents were measured by the baseline shift from the gray dotted line to the red dotted line. TTX-insensitive tonic GABA currents were measured by the baseline shift from the red dotted line to the blue dotted line. **b**, Bar graphs comparing TTX-sensitive and TTX-insensitive tonic GABA currents between the control and general GABA-T KD groups. *n* = 3 mice per group. **c**, Paired scatter plots showing the IPSC frequency (left) and IPSC amplitude (right) in the control and general GABA-T KD groups before and after TTX treatment. *n* = 3 mice per group. The data are presented as the mean ± standard error of the mean. The individual dots refer to cells, unless otherwise specified. The *P* values were obtained via two-way ANOVA (Šídák’s multiple comparisons test) for **b** and a two-way repeated-measures ANOVA (uncorrected Fisher’s LSD) for **c**. **P < 0.01, ****P < 0.0001; nonsignificant (ns) indicates a *P* > 0.05.

An alternative possibility is that suppressing GABA-T in GABAergic neurons could enhance tonic inhibition through action-potential-dependent synaptic GABA spillover. We used TTX, an inhibitor of voltage-gated sodium channels that mediate action potentials, and measured TTX-sensitive and TTX-insensitive tonic GABA currents in dentate GCs following general GABA-T KD to explore this hypothesis. We observed a significant increase in TTX-insensitive tonic GABA currents in the general GABA-T KD group compared with the control group, whereas TTX-sensitive tonic GABA currents were not statistically different between the control and general GABA-T KD groups (Fig. 5a,b). Both the control and general GABA-T KD groups presented a reduced inhibitory postsynaptic current (IPSC) frequency after TTX treatment without a change in amplitude, but the reduction was significant in the general GABA-T KD group (Fig. 5c). In summary, these findings indicate that the enhanced tonic inhibition following general GABA-T KD is mainly action-potential independent.

### Suppressing astrocytic GABA-T impairs spatial long-term memory by reducing the spike probability of dentate GCs

**Fig. 6:**
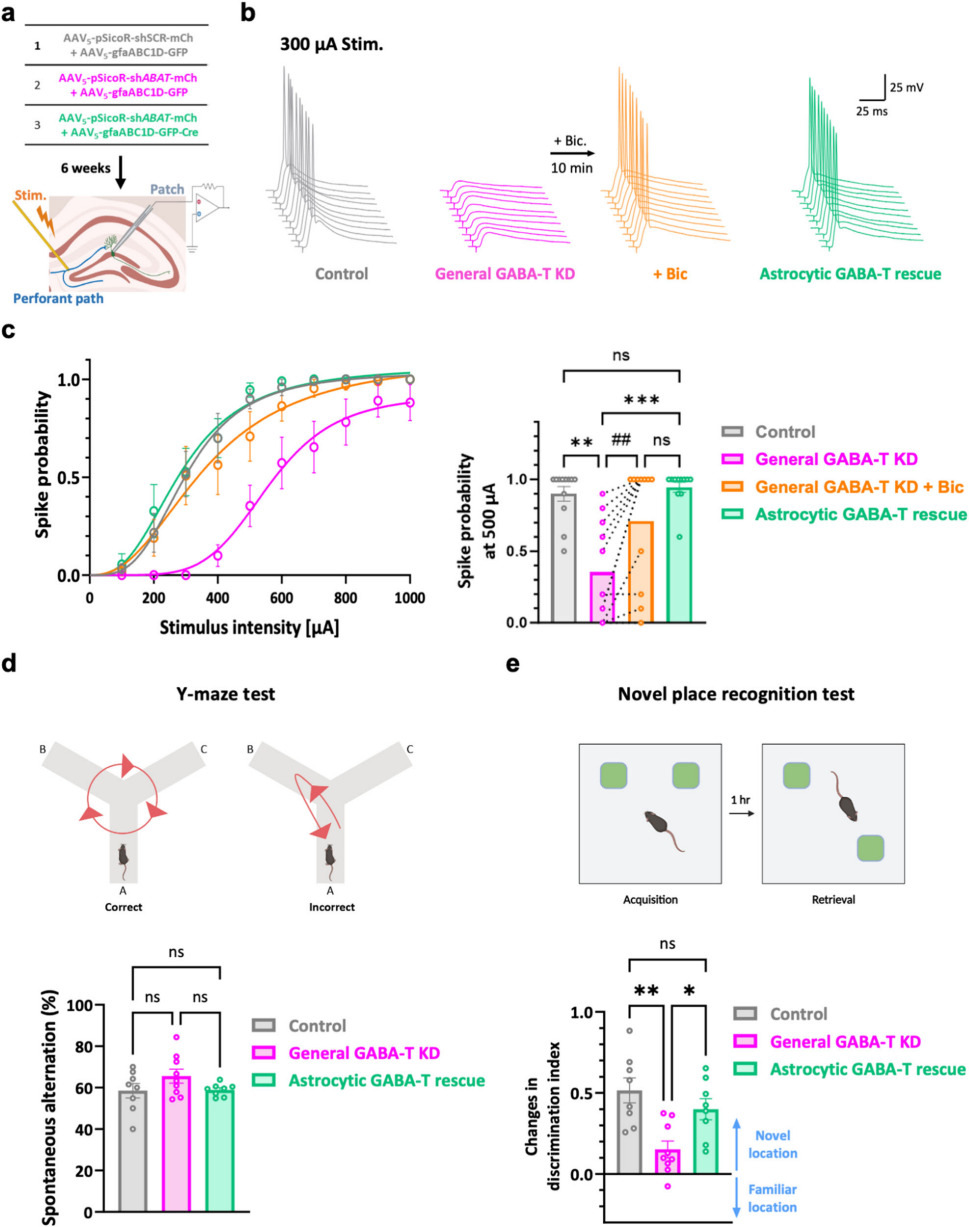
Sole suppression of astrocytic GABA-T adequately diminishes both the excitability of dentate GCs and hippocampal spatial long-term memory. **a**, A workflow illustrating the silencing of the GABA-T gene (either including or excluding astrocytes) in the dorsal hippocampal DG, followed by measurements of the evoked spike probability. **b**, Representative traces of evoked excitatory postsynaptic potentials (EPSPs) in DG GCs at 300 μA electrical stimulation of the perforant path. **c**, Left: a summary graphs showing the spike probability versus the stimulus intensity. Right: a comparison of spike probabilities at 500 μA stimulation. *n* = 4 mice per group. **d**, Top: a schematic representation of the Y-maze test. Bottom: comparative bar graphs showing the percentage of spontaneous alternations. Each individual dot represents one animal. **e**, Top: a schematic representation of the novel place recognition test. Bottom: comparative bar graphs showing changes in the discrimination index. Each individual dot represents one animal. The data are presented as the mean ± standard error of the mean. The individual dots refer to cells, unless otherwise specified. The *P* values were obtained via the Kruskal‒Wallis test for **c** (right; for the comparison marked with #, a paired *t*-test was employed) and ordinary one-way ANOVA for **d** and **e**. *P < 0.05; **P < 0.01; ^##^*P* < 0.01; ***P < 0.001; nonsignificant (ns) indicates a *P* > 0.05.

We evaluated the probability of action potentials in response to electrical stimulation of perforant pathways to examine the effects of astrocytic GABA-T inhibition on synaptically elicited action-potential firing (Fig. 6a), as previously described^10,22^. This assessment was achieved by whole-cell current-clamp recordings of the dentate GCs of the mice injected with the viruses used for astrocyte-specific GABA-T manipulation within the dorsal DG (Fig. 6a). The spike probability was diminished in the GABA-T KD group compared with that in the control group (Fig. 6b,c). Interestingly, this reduction was fully reversed by blocking GABA_A_Rs or rescuing GABA-T specifically in astrocytes (Fig. 6b,c). These results indicate that astrocytic GABA-T suppression leads to increased tonic inhibition and a subsequent reduction in neuronal excitability and synaptic transmission.

We evaluated the changes in spatial memory-related behaviors resulting from genetic manipulations of GABA-T in the dorsal DG (Supplementary Fig. 2a,b) by performing a Y-maze test to assess spatial working memory and a novel place recognition test to assess spatial long-term memory 6 weeks after the virus injection. Spatial working memory, as evaluated by the spontaneous alternation task, was not affected by genetic GABA-T manipulations (Fig. 6d). This result implies that spatial working memory may not be sensitive to changes in tonic and phasic GABA-mediated inhibition within the dorsal DG. By contrast, the novel place recognition test revealed that the general KD of GABA-T impaired spatial long-term memory, as evidenced by a considerable decrease in the discrimination index (Fig. 6e). Importantly, this impairment was fully reversed by the astrocyte-specific rescue of GABA-T (Fig. 6e). These results suggest that the enhanced tonic inhibition as a consequence of astrocytic GABA-T suppression may negatively affect spatial long-term memory. Taken together, our findings emphasize that the inhibition of astrocytic GABA-T in the dorsal DG can critically modulate spatial long-term memory without noticeably influencing spatial working memory, revealing the selective impacts of astrocytic GABA-T on these distinct memory processes.

## Discussion

Here, we described the causal relationship between the disruption of the astrocytic GABA degradation pathway and enhanced tonic inhibition. We found that suppressing GABA-T in astrocytes but not GABAergic neurons is an effective approach to increase tonic inhibition. Suppressing astrocytic GABA-T alone was sufficient to dampen the excitability of GCs in the dorsal DG and impair spatial long-term memory. Our study suggests that cognitive functions could be dynamically regulated by altering GABA clearance in the brain via astrocytic GABA-T.

While enzymes associated with the production of astrocytic GABA can control neuronal excitability, our findings reveal another notable and opposing mechanism in which the degradation of astrocytic GABA also plays a crucial role in regulating neuronal excitability. Mechanistically, tonic GABA release from astrocytes can reduce the membrane resistance of neurons through the prolonged opening of extrasynaptic GABA_A_Rs, thereby reducing the excitatory postsynaptic potential and neuronal excitability^17,32^. One approach to enhancing astrocytic tonic GABA release is the overexpression of astrocytic MAO-B^13,27^. However, this approach may also increase the amount of H_2_O_2_ as a byproduct of the MAO-B reaction, which might inadvertently result in neurodegeneration^18^. Our study proposes to address this issue by suppressing astrocytic GABA-T to increase astrocytic tonic GABA release without unwanted side effects such as increased H_2_O_2_ production.

Unlike physiological conditions, we used an internal solution with a high Cl^−^ concentration to examine the effects of astrocytic GABA-T suppression on neuronal firing. Under these experimental conditions, the GABA_A_ current reversal potential (*E*_GABA_) was depolarizing, rendering the baseline tonic GABA_A_ conductance excitatory. However, as astrocytic GABA-T suppression substantially increased tonic GABA_A_ conductance, the initial excitatory effect of depolarization appeared to eventually be overridden by inhibition^55^. This shift can be explained by shunting inhibition, whereby the increase in tonic GABA_A_ conductance diminishes the effectiveness of excitatory inputs through increased membrane conductance. Consequently, astrocytic GABA-T suppression probably increases ambient GABA to levels where shunting inhibition dominates, even with a depolarizing *E*_GABA_, thereby decreasing the spike probability. This experimental setup underscores the robustness of shunting inhibition mediated by astrocytic GABA-T suppression in modulating neuronal excitability.

Interestingly, both GABA-T and SSADH, which are located in the mitochondria, exhibit higher mRNA expression in astrocytes than in other brain cells^56–58^. Given the augmented tonic inhibition observed in SSADH-knockout mice^48,49^, astrocytic GABA-T and astrocytic SSADH may have similar roles in modulating tonic inhibition. Thus, our findings with astrocytic GABA-T should provide a relevant framework for understanding the potential role of astrocytic SSADH. GABA needs to be taken up into the mitochondria to be metabolized by GABA-T and SSADH. However, the specific mechanism by which GABA is transported into the mitochondria is not yet clear. Assuming the presence of a GABA transporter within the mitochondrial membrane, the inhibition of this transporter could enhance astrocytic tonic GABA release. This exciting possibility warrants future investigations.

The importance of glial GABA-T in brain function has been highlighted in a sleep study using a *Drosophila* model, which revealed that a loss of glial GABA-T increases both the daily sleep duration and sleep consolidation, potentially through increased tonic GABA release from glial cells^59^. In terms of cognitive functions, we have shown that astrocytic GABA-T suppression in the dorsal DG impaired long-term spatial memory. However, spatial working memory was intact even after genetic GABA-T modulations. This pattern of cognitive effects is similar to the findings of previous studies, which reported that nectin-3 KD in the dorsal DG specifically disrupted long-term spatial memory without altering spatial working memory^60,61^. Spatial working memory may rely more on other brain regions, such as the medial prefrontal cortex (mPFC) and ventral hippocampus, which have been identified as crucial regions for the encoding of spatial working memory^62–66^. As the GABA-T KD was confined to the dorsal hippocampus in our study, it may not have impacted the functionality of the mPFC and ventral hippocampus, thereby leaving spatial working memory undamaged. However, further studies are needed to fully elucidate the distinct physiological roles of astrocytic GABA-T in other brain regions, including the mPFC and ventral hippocampus.

Changes in GABA-T expression levels have been explored in subjects with various neuropathological conditions. For example, pathological aggressive behavior in BALB/cJ mice is associated with a 40% reduction in ^1^H-MRS GABA levels and a 20-fold increase in *ABAT* expression in the ventral anterior cingulate cortex compared with those in BALB/cByJ mice^67^. Another study revealed that the mRNA expression of *ABAT* and *ALDH5A1* was markedly decreased in the striatum, hippocampus and cerebellum of rats with depression induced by chronic unpredictable mild stress^68^. Investigating whether variations in astrocytic GABA-T expression levels contribute to these neuropsychiatric disorders could be enlightening, especially through targeted overexpression or gene silencing of GABA-T in astrocytes in different brain regions. Our research should provide insights into such pathogenic mechanisms.

Our AAV-based astrocytic GABA-T inhibition could be applied in a brain region-specific manner to address the imbalance between excitation and inhibition. However, the dual nature of astrocytic GABA-T inhibition must be considered. Although the inhibition of GABA-T in astrocytes shows promise in reducing neuronal excitability in the dorsal DG, our findings indicate a potential trade-off with spatial memory function. This paradox underscores the complexity of developing therapeutic strategies targeting astrocytic GABA-T. Therefore, carefully weighing the benefits of reduced neuronal excitability against the potential risks of side effects is essential.

Our findings indicate that targeted suppression of astrocytic GABA-T not only enhances tonic GABA release from astrocytes but also influences the excitatory/inhibitory balance in the brain, with consequential effects on behavior. These findings suggest that astrocytic GABA-T modulation holds promising potential for the development of novel therapeutic strategies aimed at treating cognitive and neurological disorders through the regulation of astrocytic GABA metabolism.

## Supplementary information

Supplementary figures

## References

[1] Kaneda, M., Farrant, M. & Cull-Candy, S. G. Whole‐cell and single‐channel currents activated by GABA and glycine in granule cells of the rat cerebellum. *J. Physiol.* **485**, 419–435 (1995). 7545231 10.1113/jphysiol.1995.sp020739 PMC1158002

[2] Nusser, Z., Roberts, J., Baude, A., Richards, J. G. & Somogyi, P. Relative densities of synaptic and extrasynaptic GABA_A_ receptors on cerebellar granule cells as determined by a quantitative immunogold method. *J. Neurosci.* **15**, 2948–2960 (1995). 7722639 10.1523/JNEUROSCI.15-04-02948.1995 PMC6577757

[3] Semyanov, A., Walker, M. C., Kullmann, D. M. & Silver, R. A. Tonically active GABA_A_ receptors: modulating gain and maintaining the tone. *Trends Neurosci.* **27**, 262–269 (2004). 15111008 10.1016/j.tins.2004.03.005

[4] Hamann, M., Rossi, D. J. & Attwell, D. Tonic and spillover inhibition of granule cells control information flow through cerebellar cortex. *Neuron* **33**, 625–633 (2002). 11856535 10.1016/s0896-6273(02)00593-7

[5] Farrant, M. & Nusser, Z. Variations on an inhibitory theme: phasic and tonic activation of GABA_A_ receptors. *Nat. Rev. Neurosci.* **6**, 215–229 (2005). 15738957 10.1038/nrn1625

[6] Jia, F. et al. An extrasynaptic GABA_A_ receptor mediates tonic inhibition in thalamic VB neurons. *J. Neurophysiol.* **94**, 4491–4501 (2005). 16162835 10.1152/jn.00421.2005

[7] Caraiscos, V. B. et al. Tonic inhibition in mouse hippocampal CA1 pyramidal neurons is mediated by α5 subunit-containing γ-aminobutyric acid type A receptors. *Proc. Natl Acad. Sci. USA* **101**, 3662–3667 (2004). 14993607 10.1073/pnas.0307231101 PMC373519

[8] Yoon, B.-E. & Lee, C. J. GABA as a rising gliotransmitter. *Front. Neural Circuits* **8**, 141 (2014). 25565970 10.3389/fncir.2014.00141 PMC4269106

[9] Mody, I. & Pearce, R. A. Diversity of inhibitory neurotransmission through GABA_A_ receptors. *Trends Neurosci.* **27**, 569–575 (2004). 15331240 10.1016/j.tins.2004.07.002

[10] Jo, S. et al. GABA from reactive astrocytes impairs memory in mouse models of Alzheimer’s disease. *Nat. Med.* **20**, 886–896 (2014). 24973918 10.1038/nm.3639 PMC8385452

[11] Kim, Y. S., Woo, J., Lee, C. J. & Yoon, B.-E. Decreased glial GABA and tonic inhibition in cerebellum of mouse model for attention-deficit/hyperactivity disorder (ADHD). *Exp. Neurobiol.* **26**, 206 (2017). 28912643 10.5607/en.2017.26.4.206 PMC5597551

[12] Chun, H. et al. Astrocytic proBDNF and tonic GABA distinguish active versus reactive astrocytes in hippocampus. *Exp. Neurobiol.* **27**, 155 (2018). 30022867 10.5607/en.2018.27.3.155 PMC6050417

[13] Woo, J. et al. Control of motor coordination by astrocytic tonic GABA release through modulation of excitation/inhibition balance in cerebellum. *Proc. Natl Acad. Sci. USA* **115**, 5004–5009 (2018). 29691318 10.1073/pnas.1721187115 PMC5948981

[14] Park, J.-H. et al. Newly developed reversible MAO-B inhibitor circumvents the shortcomings of irreversible inhibitors in Alzheimer’s disease. *Sci. Adv.* **5**, eaav0316 (2019). 30906861 10.1126/sciadv.aav0316 PMC6426469

[15] Heo, J. Y. et al. Aberrant tonic inhibition of dopaminergic neuronal activity causes motor symptoms in animal models of Parkinson’s disease. *Curr. Biol.* **30**, 276–291. e279 (2020). 31928877 10.1016/j.cub.2019.11.079

[16] Nam, M. H. et al. Excessive astrocytic GABA causes cortical hypometabolism and impedes functional recovery after subcortical stroke. *Cell Rep*. **32**, 107861 (2020). 32640227 10.1016/j.celrep.2020.107861

[17] Kwak, H. et al. Astrocytes control sensory acuity via tonic inhibition in the thalamus. *Neuron* **108**, 691–706. e610 (2020). 32905785 10.1016/j.neuron.2020.08.013

[18] Chun, H. et al. Severe reactive astrocytes precipitate pathological hallmarks of Alzheimer’s disease via H_2_O_2_^−^ production. *Nat. Neurosci.* **23**, 1555–1566 (2020). 33199896 10.1038/s41593-020-00735-y

[19] Srivastava, I., Vazquez-Juarez, E., Henning, L., Gómez-Galán, M. & Lindskog, M. Blocking astrocytic GABA restores synaptic plasticity in prefrontal cortex of rat model of depression. *Cells* **9**, 1705 (2020). 32708718 10.3390/cells9071705 PMC7408154

[20] Nam, M.-H. et al. KDS2010, a newly developed reversible MAO-B inhibitor, as an effective therapeutic candidate for Parkinson’s disease. *Neurotherapeutics* **18**, 1729–1747 (2021). 34611843 10.1007/s13311-021-01097-4 PMC8608967

[21] Won, W. et al. Inhibiting peripheral and central MAO-B ameliorates joint inflammation and cognitive impairment in rheumatoid arthritis. *Exper. Mol. Med.* **54**, 1188–1200 (2022). 35982301 10.1038/s12276-022-00830-z PMC9440195

[22] Ju, Y. H. et al. Astrocytic urea cycle detoxifies Aβ-derived ammonia while impairing memory in Alzheimer’s disease. *Cell Metabolism* **34**, 1104–1120. e1108 (2022). 35738259 10.1016/j.cmet.2022.05.011

[23] Cheng, Y.-T. et al. Social deprivation induces astrocytic TRPA1-GABA suppression of hippocampal circuits. *Neuron* **111**, 1301–1315. e1305 (2023). 36787749 10.1016/j.neuron.2023.01.015 PMC10121837

[24] Sa, M. et al. Hypothalamic GABRA5-positive neurons control obesity via astrocytic GABA. *Nat. Metab.* **5**, 1506–1525 (2023). 37653043 10.1038/s42255-023-00877-w

[25] Yoon, B. E. et al. Glial GABA, synthesized by monoamine oxidase B, mediates tonic inhibition. *J. Physiol.* **592**, 4951–4968 (2014). 25239459 10.1113/jphysiol.2014.278754 PMC4259537

[26] Lee, N., Sa, M., Hong, Y. R., Lee, C. J. & Koo, J. Fatty acid increases cAMP-dependent lactate and MAO-B-dependent GABA production in mouse astrocytes by activating a Gαs protein-coupled receptor. *Exper. Neurobiol.* **27**, 365 (2018). 30429646 10.5607/en.2018.27.5.365 PMC6221839

[27] An, H., Heo, J. Y., Lee, C. J. & Nam, M.-H. The pathological role of astrocytic MAOB in Parkinsonism revealed by genetic ablation and over-expression of MAOB. *Exper. Neurobiol.* **30**, 113 (2021). 33972465 10.5607/en21007 PMC8118757

[28] Cho, H.-U. et al. Redefining differential roles of MAO-A in dopamine degradation and MAO-B in tonic GABA synthesis. *Exper. Mol. Med.* **53**, 1148–1158 (2021). 34244591 10.1038/s12276-021-00646-3 PMC8333267

[29] Chun, H., Lim, J., Park, K. D. & Lee, C. J. Inhibition of monoamine oxidase B prevents reactive astrogliosis and scar formation in stab wound injury model. *Glia* **70**, 354–367 (2022). 34713936 10.1002/glia.24110

[30] Lee, J. M. et al. Generation of astrocyte-specific MAOB conditional knockout mouse with minimal tonic GABA inhibition. *Exper. Neurobiol.* **31**, 158 (2022). 35786639 10.5607/en22016 PMC9272118

[31] Nam, M.-H., Sa, M., Ju, Y. H., Park, M. G. & Lee, C. J. Revisiting the role of astrocytic MAOB in Parkinson’s disease. *Int. J. Mol. Sci.* **23**, 4453 (2022). 35457272 10.3390/ijms23084453 PMC9028367

[32] Koh, W., Kwak, H., Cheong, E. & Lee, C. J. GABA tone regulation and its cognitive functions in the brain. *Nat. Rev. Neurosci.* **24**, 523–539 (2023). 37495761 10.1038/s41583-023-00724-7

[33] Lee, S. et al. Channel-mediated tonic GABA release from glia. *Science* **330**, 790–796 (2010). 20929730 10.1126/science.1184334

[34] Pandit, S. et al. Bestrophin1‐mediated tonic GABA release from reactive astrocytes prevents the development of seizure‐prone network in kainate‐injected hippocampi. *Glia* **68**, 1065–1080 (2020). 31833596 10.1002/glia.23762

[35] Egawa, K. & Fukuda, A. Pathophysiological power of improper tonic GABA_A_ conductances in mature and immature models. *Front. Neural Circuits* **7**, 170 (2013). 24167475 10.3389/fncir.2013.00170 PMC3807051

[36] Olsen, M. L. et al. New insights on astrocyte ion channels: critical for homeostasis and neuron-glia signaling. *J. Neurosci.* **35**, 13827–13835 (2015). 26468182 10.1523/JNEUROSCI.2603-15.2015 PMC4604221

[37] Oh, S.-J. & Lee, C. J. Distribution and function of the bestrophin-1 (Best1) channel in the brain. *Exper. Neurobiol.* **26**, 113 (2017). 28680296 10.5607/en.2017.26.3.113 PMC5491579

[38] Park, M. G. et al. High-yield synthesis and purification of recombinant human GABA transaminase for high-throughput screening assays. *J. Enzyme Inhib. Med. Chem.* **36**, 2016–2024 (2021). 34514924 10.1080/14756366.2021.1975697 PMC8439235

[39] Hsu, Y.-T., Chang, Y.-G. & Chern, Y. Insights into GABA_A_ergic system alteration in Huntington’s disease. *R. Soc. Open Biol.* **8**, 180165 (2018). 10.1098/rsob.180165 PMC6303784 30518638

[40] Pan, Y. et al. CPP-115, a potent γ-aminobutyric acid aminotransferase inactivator for the treatment of cocaine addiction. *J. Med. Chem.* **55**, 357 (2012). 22128851 10.1021/jm201231w PMC3257419

[41] Silverman, R. B. The 2011 EB Hershberg Award for important discoveries in medicinally active substances:(1*S*, 3*S*)-3-amino-4-difluoromethylenyl-1-cyclopentanoic acid (CPP-115), a GABA aminotransferase inactivator and new treatment for drug addiction and infantile spasms. *J. Med. Chem.* **55**, 567–575 (2012). 22168767 10.1021/jm201650r PMC3266980

[42] Doumlele, K., Conway, E., Hedlund, J., Tolete, P. & Devinsky, O. A case report on the efficacy of vigabatrin analogue (1*S*, 3*S*)-3-amino-4-difluoromethylenyl-1-cyclopentanoic acid (CPP-115) in a patient with infantile spasms. *Epilepsy Beh. Case Rep.* **6**, 67–69 (2016). 10.1016/j.ebcr.2016.08.002 PMC5024311 27668180

[43] Silverman, R. B. Design and mechanism of GABA aminotransferase inactivators. Treatments for epilepsies and addictions. *Chem. Rev.* **118**, 4037–4070 (2018). 29569907 10.1021/acs.chemrev.8b00009 PMC8459698

[44] Feja, M. et al. OV329, a novel highly potent γ‐aminobutyric acid aminotransferase inactivator, induces pronounced anticonvulsant effects in the pentylenetetrazole seizure threshold test and in amygdala‐kindled rats. *Epilepsia* **62**, 3091–3104 (2021). 34617595 10.1111/epi.17090 PMC8639636

[45] Bialer, M. et al. Progress report on new antiepileptic drugs: a summary of the Sixteenth Eilat Conference on New Antiepileptic Drugs and Devices (EILAT XVI): II. Drugs in more advanced clinical development. *Epilepsia* **63**, 2883–2910 (2022). 35950617 10.1111/epi.17376

[46] Wu, Y., Wang, W. & Richerson, G. B. GABA transaminase inhibition induces spontaneous and enhances depolarization-evoked GABA efflux via reversal of the GABA transporter. *J. Neurosci.* **21**, 2630–2639 (2001). 11306616 10.1523/JNEUROSCI.21-08-02630.2001 PMC6762542

[47] Wu, Y., Wang, W. & Richerson, G. B. Vigabatrin induces tonic inhibition via GABA transporter reversal without increasing vesicular GABA release. *J. Neurophysiol.* **89**, 2021–2034 (2003). 12612025 10.1152/jn.00856.2002

[48] Drasbek, K., Vardya, I., Delenclos, M., Gibson, K. & Jensen, K. SSADH deficiency leads to elevated extracellular GABA levels and increased GABAergic neurotransmission in the mouse cerebral cortex. *J. Inherit. Metab. Disease* **31**, 662–668 (2008). 18696252 10.1007/s10545-008-0941-7 PMC2596865

[49] Errington, A. C., Gibson, K. M., Crunelli, V. & Cope, D. W. Aberrant GABA_A_ receptor-mediated inhibition in cortico-thalamic networks of succinic semialdehyde dehydrogenase deficient mice. *PloS ONE* **6**, e19021 (2011). 21526163 10.1371/journal.pone.0019021 PMC3079762

[50] Nanavati, S. M. & Silverman, R. B. Mechanisms of inactivation of γ-aminobutyric acid aminotransferase by the antiepilepsy drug γ-vinyl GABA (vigabatrin). *J. Am. Chem. Soc.* **113**, 9341–9349 (1991).

[51] Lee, C. J. & Yoon, B.-E. Protease-activated receptor 1-induced GABA release in cultured cortical astrocytes pretreated with GABA is mediated by the Bestrophin-1 channel. *Anim. Cells Syst.* **18**, 244–249 (2014).

[52] Lee, M., McGeer, E. G. & McGeer, P. L. Mechanisms of GABA release from human astrocytes. *Glia* **59**, 1600–1611 (2011). 21748804 10.1002/glia.21202

[53] Ventura, A. et al. Cre-lox-regulated conditional RNA interference from transgenes. *Proc. Natl Acad. Sci.* **101**, 10380–10385 (2004). 15240889 10.1073/pnas.0403954101 PMC478580

[54] Lee, Y., Messing, A., Su, M. & Brenner, M. GFAP promoter elements required for region‐specific and astrocyte‐specific expression. *Glia* **56**, 481–493 (2008). 18240313 10.1002/glia.20622

[55] Song, I., Savtchenko, L. & Semyanov, A. Tonic excitation or inhibition is set by GABA_A_ conductance in hippocampal interneurons. *Nat. Commun.* **2**, 376 (2011). 21730957 10.1038/ncomms1377 PMC3144593

[56] Chai, H. et al. Neural circuit-specialized astrocytes: transcriptomic, proteomic, morphological, and functional evidence. *Neuron* **95**, 531–549. e539 (2017). 28712653 10.1016/j.neuron.2017.06.029 PMC5811312

[57] Srinivasan, R. et al. New transgenic mouse lines for selectively targeting astrocytes and studying calcium signals in astrocyte processes in situ and in vivo. *Neuron* **92**, 1181–1195 (2016). 27939582 10.1016/j.neuron.2016.11.030 PMC5403514

[58] Schousboe, A., Bak, L. K. & Waagepetersen, H. S. Astrocytic control of biosynthesis and turnover of the neurotransmitters glutamate and GABA. *Front. Endocrinol.* **4**, 102 (2013). 10.3389/fendo.2013.00102 PMC3744088 23966981

[59] Chen, W.-F. et al. A neuron–glia interaction involving GABA transaminase contributes to sleep loss in sleepless mutants. *Mol. Psychiatry* **20**, 240–251 (2015). 24637426 10.1038/mp.2014.11 PMC4168011

[60] Wang, X.-D. et al. Nectin-3 links CRHR1 signaling to stress-induced memory deficits and spine loss. *Nat. Neurosci.* **16**, 706–713 (2013). 23644483 10.1038/nn.3395

[61] Wang, X. et al. Nectin-3 modulates the structural plasticity of dentate granule cells and long-term memory. *Transl. Psychiatry* **7**, e1228–e1228 (2017). 28872640 10.1038/tp.2017.196 PMC5639241

[62] O’Neill, P.-K., Gordon, J. A. & Sigurdsson, T. Theta oscillations in the medial prefrontal cortex are modulated by spatial working memory and synchronize with the hippocampus through its ventral subregion. *J. Neurosci.* **33**, 14211–14224 (2013). 23986255 10.1523/JNEUROSCI.2378-13.2013 PMC3756763

[63] Spellman, T. et al. Hippocampal–prefrontal input supports spatial encoding in working memory. *Nature* **522**, 309–314 (2015). 26053122 10.1038/nature14445 PMC4505751

[64] Tamura, M., Spellman, T. J., Rosen, A. M., Gogos, J. A. & Gordon, J. A. Hippocampal-prefrontal theta-gamma coupling during performance of a spatial working memory task. *Nat. Commun.* **8**, 2182 (2017). 29259151 10.1038/s41467-017-02108-9 PMC5736608

[65] Xia, M., Liu, T., Bai, W., Zheng, X. & Tian, X. Information transmission in HPC-PFC network for spatial working memory in rat. *Behav. Brain Res.* **356**, 170–178 (2019). 30170031 10.1016/j.bbr.2018.08.024

[66] Salimi, M., Tabasi, F., Nazari, M., Ghazvineh, S. & Raoufy, M. R. The olfactory bulb coordinates the ventral hippocampus–medial prefrontal cortex circuit during spatial working memory performance. *J. Physiol. Sci.* **72**, 9 (2022). 35468718 10.1186/s12576-022-00833-5 PMC10717655

[67] Jager, A. et al. Cortical control of aggression: GABA signalling in the anterior cingulate cortex. *Eur. Neuropsychopharmacol.* **30**, 5–16 (2020). 29274996 10.1016/j.euroneuro.2017.12.007

[68] Xu, S. et al. Chronic stress in a rat model of depression disturbs the glutamine–glutamate–GABA cycle in the striatum, hippocampus, and cerebellum. *Neuropsychiatr*. **16**, 557–570 (2020). 10.2147/NDT.S245282 PMC7047974 32158215

