# Supplementary material for: Suppressing astrocytic GABA transaminase enhances tonic inhibition and weakens hippocampal spatial memory
Source: Exp Mol Med. 2025 Feb 3;57(2):379–89. doi: 10.1038/s12276-025-01398-0 (PMC11873293; doi:10.1038/s12276-025-01398-0)
Supplement: Supplementary file 1 — Supplementary figures [file 12276_2025_1398_MOESM1_ESM.pdf]

Supplementary Fig. 1

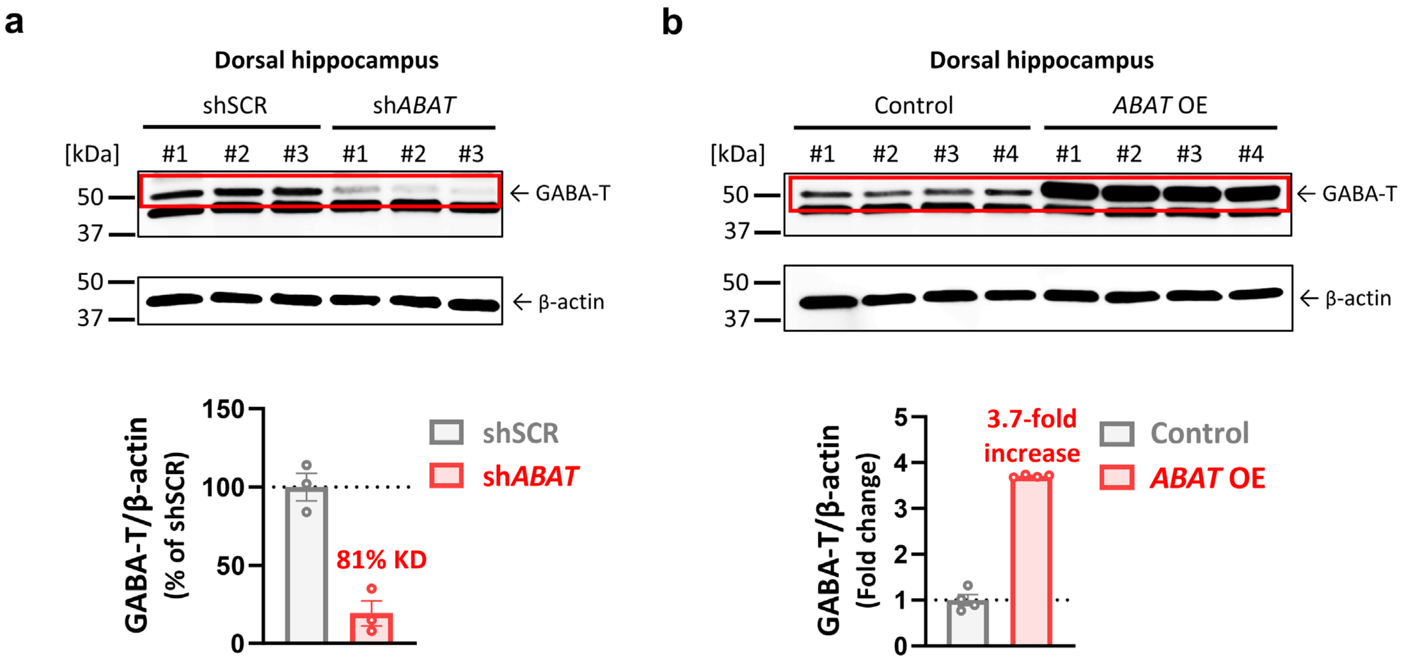

Supplementary Fig. 1 Confirmation of GABA-T knockdown (KD) and overexpression (OE) efficiency in dorsal hippocampal tissue by western blot analysis.

a Top: **w**estern blot analysis of GABA-T and β-actin-immunoreactive proteins from mouse dorsal hippocampal tissue infected with either AAV_5-pSicoR-shSCR-mCherry or AAV_5-pSicoR-sh*ABAT*-mCherry. Bottom: comparative bar graphs representing the expression ratio of GABA-T/ β-actin. Each dot represents an individual mouse.

b Top: **w**estern blot analysis of GABA-T and β-actin-immunoreactive proteins from mouse dorsal hippocampal tissue infected with either AAV_5-gfaABC1D-GFP or AAV_5-gfaABC1D-m*ABAT*-2A-GFP. Bottom: comparative bar graphs representing the expression ratio of GABA-T/ β-actin. Each dot represents an individual mouse.

Supplementary Fig. 2

a

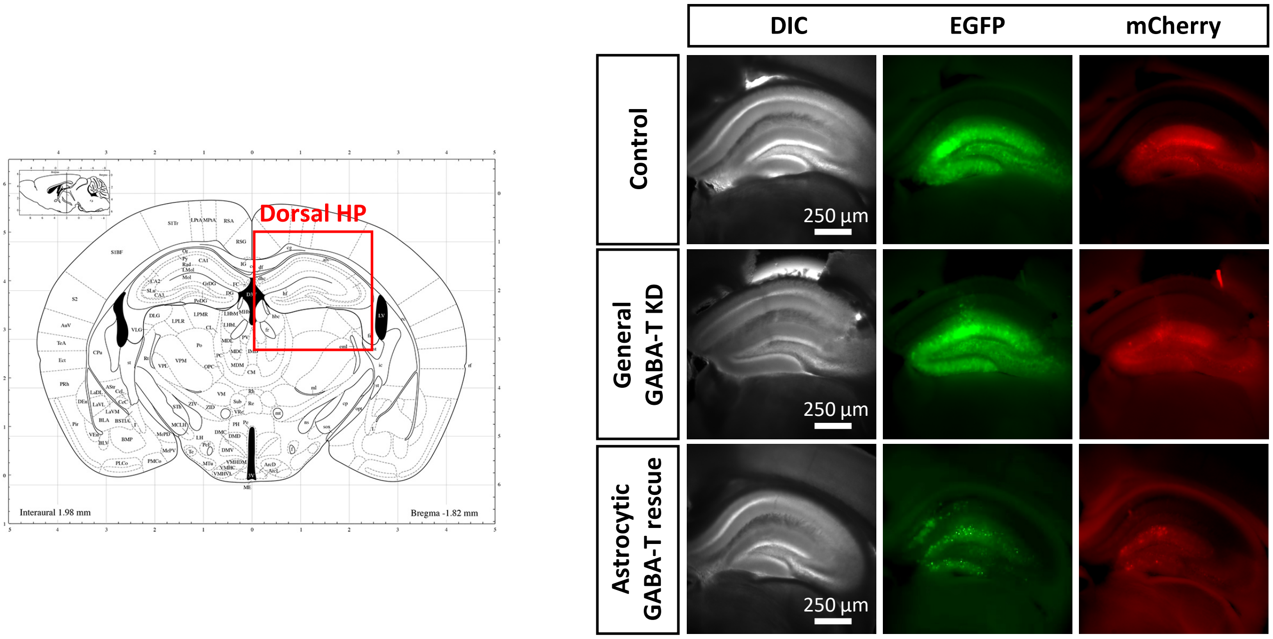

b

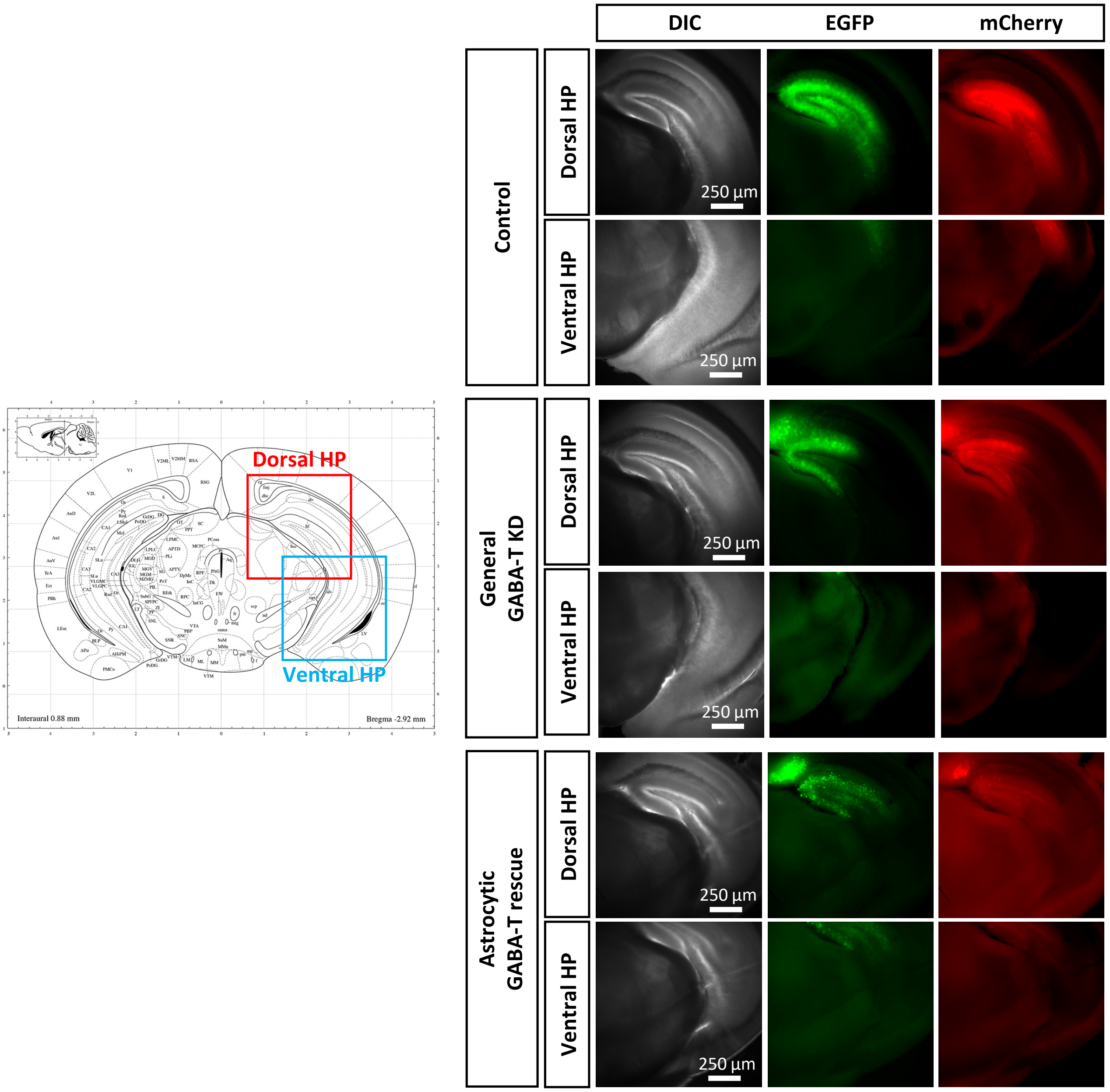

Supplementary Fig. 2 The distribution of GABA-T KD and astrocytic rescue viruses in the brains of mice used for behavioral experiments.

a Representative wide field images of coronal sections including the dorsal hippocampus (HP), showing the distribution of viruses in the dorsal dentate gyrus.

b Representative wide field images of coronal sections including both the dorsal and ventral HP, showing the distribution of viruses in the dorsal dentate gyrus.
